# Does Cup-Grip Type Affect Tremor among People with Essential Tremor?

**DOI:** 10.3390/s21237797

**Published:** 2021-11-23

**Authors:** Navit Roth, Sara Rosenblum

**Affiliations:** 1 The Laboratory of Complex Human Activity and Participation (CHAP), Department of Occupational Therapy, University of Haifa, Haifa 3498838, Israel; rosens@research.haifa.ac.il; 2 Department of Mechanical Engineering, ORT Braude Academic College of Engineering, Karmiel 2161002, Israel

**Keywords:** essential tremor, cup, grip type

## Abstract

Essential tremor (ET) is a movement disorder that may cause functional disability in daily activities, such as drinking from a cup or drawing. This study aims to characterize effects of varied cup-grip types and measured axes on the actual performance of people with ET and find correlations between cup-grip type and measured axes, and spiral drawing measures. Participants (20 with ET and 18 controls) held a cup of water in a steady position in three grip types and drew a spiral. The cup acceleration was measured by the cup triaxial accelerometer, analyzed in *X*, *Y* and *Z* axes (directions); deviation of the measured acceleration from the desired steady position acceleration was computed. Significant group differences were found for outcome measures in all grip types. Among participants with ET, significantly higher measured values were found in the cup’s horizontal plane (*X* and *Y* axes) compared to the vertical direction (*Z* axis) and for on-the-handle versus around-the-cup grips in the *X* and *Y* axes. Significant correlations were found between this grip’s measures and spiral-drawing actual performance measures, indicating the measurement axis and grip type may affect actual performance. These findings may support the future development of assistive devices for tremor suppression and personalized supportive therapy.

## 1. Introduction

Essential tremor (ET) is a common movement disorder characterized mainly as an action tremor of the upper limbs [1,2,3]. The tremor may affect functional ability in the performance of activities of daily living (ADL), such as writing, using a spoon, or drinking from, holding, or carrying a cup, and this may impact quality of life [4,5].

Assessing tremor effect on functional ability may be performed using two main methods. The first is self-reported disability questionnaires, and the second is performance-based tests scored by a rater, which may include objective measurement systems [6,7,8,9]. Quantifying the tremor effect in various ADL tasks using measurement systems at the clinic may provide valuable data, including characteristics such as amplitude and frequency. Such quantification systems include digital graphic boards, electromyography, and accelerometers [10]. Previous research using such measurement systems included various ADL tasks, such as writing; drawing; pouring, drinking from, or holding a cup; using a spoon, computer mouse, keyboard, or remote control; and folding laundry [7,9,11,12,13,14]. Some research implemented accelerometer measurement tools to evaluate the tools’ ability to characterize the tremor and its severity [11], or to validate tremor scales [7], whereas others used graphical boards to evaluate the effects of task characteristics such as drawing direction [15].

Using acceleration sensors is one of the most widely studied methods for measuring tremor characteristics. Data analysis of acceleration signals includes integration to compute velocity and displacement, as well as frequency and amplitude measures using both time- and frequency-domain methods [10,16,17,18].

Our current study is a part of a larger research, the main goal of which was to produce objective measures of ADL task-performance characteristics in order to assess tremor effects on functional ability in ET. Further, it compared tremor effects on functionality to that of controls to determine measures sensitive to that effect among people with ET. The first part of the study addressed self-reported disability caused by ET and modifications that participants with ET implemented to decrease the tremor [19], using the Columbia University Assessment of Disability in Essential Tremor (CADET) disability questionnaire [6,7]. The second part addressed the effect of drawing direction on task-performance characteristics while drawing lines and spirals on a computer digitizer [15] among participants with ET compared to controls. In the research reported herein, we focused on the effects of the task characteristics on a chosen daily task. Specifically, we analyze effects of grip types on acceleration in a cup-holding task.

Cup tasks are ADLs that may enable for assessing the effect of tremor on functional ability. Drinking from, holding, and carrying cups are among the most prevalent ADLs that ET participants report in disability questionnaires [4,5]. Some ET performance-based tests, such as those presented in the CADET, include tasks involving drinking and carrying cups [6,7]. Drinking and cup-carrying tasks are also included in ADL questionnaires, such as the CADET disability questionnaire [8]. A cup-holding task is also included in the Bain and Findley Tremor ADL Scale [9]. Gironell and colleagues suggested a self-reported drinking task (e.g., the Glass Scale) [20] as a tool to assess tremor severity. In this tool, participants are asked how they drink from a glass; whether it is difficult and whether they fill their cup with less liquid, use both hands, or use a straw [20].

By implementing the quantification systems of accelerometers in drinking tasks, significant correlations have been found between power-spectrum measures calculated from accelerometer data and clinicians’ tremor-assessment outcome measures [11]. Regarding the cup-holding task, significant correlations have been found among the ET group for the volume of spilled water and the acceleration measurements of postural hand tremors [21].

Previous research mainly investigated acceleration measurements of tremulous hands during writing, drawing, hand-posture, or other tasks, rather than the acceleration of the objects held while performing ADL tasks [13,22,23,24]. Regarding holding, drinking from, and carrying cups, the tremor measurements in previous studies included the volume of water spilled from the cup and the acceleration of the hand while holding a filled cup [12,21]. However, our review of the literature revealed no detailed data on cup acceleration of varied grip types in cup-holding tasks.

In fact, a variety of grip types and prehension can be performed while holding an object [25,26]. Grip types may be affected by the object’s (e.g., cup’s) varied designs, such as size or handles. These variations can result in changed task characteristics, such as hand posture and applied forces. Because the tremor manifests in different joints and directions of the upper limb [27], the grip type while holding a cup may affect the tremor and the ability to hold the cup steady. For instance, researchers showed that arm support did not affect posture tremors [23] and, although the weight loaded on the extended hand in posture tasks may not affect tremor frequency [22], it may affect tremor amplitude [28]. Golan and colleagues [12] found that hand position and posture (relative to the chair armrest and mouth) affected tremor frequency in one subgroup of ET participants in holding a filled cup task. Further, tremor amplitude was highest while holding the cup in the position near the mouth [12].

Our literature review also revealed no detailed results regarding the effect of grip type on the cup- or hand-acceleration measurements or a comparison between ET and control groups for different grip types. Thus, in our research, we measured cup instability from the acceleration of the cup itself (not the hand). Holding the cup in a steady position requires minimal changes in acceleration; thus, we defined and analyzed cup-acceleration measures. Because tremor manifests in different joint directions [27], and the functional outcome (actual performance axis) can be identified in other ADL tasks such as drawing (spiral main axis) [29], we explored the differences between axis acceleration while investigating the effect of cup-grip types on tremor characteristics.

Results from previous studies showed that the tremor amplitude and frequency in ET may change among ADLs, such as from drawing to posture tasks [13,14,21]. Past research also implemented spiral tasks to measure and assess actual performance among people with ET [9,30] and found significant correlations between the ET group for the volume of water spilled in cup-holding tasks and the raters’ spiral scores [21]. Analyzing the relationship between cup-grip type and tasks such as drawing may help to better understand how tremor manifests differently during ADL-task performance. Thus, to broaden the data regarding tremor effect on ADL-task performance, we included correlation analysis between cup-acceleration measures in varied grip types of cup-holding tasks (cup stability) and drawing tasks (pen-tip stability) characteristics as manifested by deviations of drawn curves from filtered curves.

Addressing tremor-axis grip-type effects and their relationship to other drawing tasks may help widen the theoretical base for the design of assistive devices for tremor suppression. In addition, it may help to better understand the effect of biomechanical characteristics and, from a personalized medical perspective, whether each person coping with ET has a preferred grip type.

Thus, this study’s aims were to: (1) map the actual performance of cup-holding tasks with varied grip types, comparing ET participants to controls, (2) map differences between axes (directions) of cup acceleration in ET participants, (3) analyze the effects of grip type on cup acceleration in ET participants, and (4) analyze correlations between actual performance measures of the cup and spiral drawing tasks, as well as with other reported demographic and disability data.

## 2. Methods

### 2.1. Participants

This study included 38 participants: 20 in the ET group, and 18 in the control group. Participants were at least 20 years old and recruited from the general population by advertisements in social media and bulletin boards. Participants in the ET and control group were the same sample used in previous studies [15,19] (one study without the control group [19]).

The ET-group inclusion criteria required presenting documentation of an ET diagnosis and, to the best of the applicant’s knowledge, having no other cause for the tremor. The control group inclusion criteria required having no tremor or other condition that could cause a hand disability to the best of their knowledge. All participants had no cognitive deterioration based on the Mini-Mental State Examination (MMSE) questionnaires [31]. Participants in both groups were matched for age and gender. The University of Haifa Ethics Committee approved this research (Approval no. 018/19), and all participants signed informed consent forms.

### 2.2. Research Instruments

#### 2.2.1. Questionnaires

Participants from both groups completed a demographic, general information, and MMSE questionnaire [31]. Participants from the ET group also completed the CADET disability questionnaire [8].

#### 2.2.2. Performance-Based Tasks

Performance-based tasks included cup-holding and spiral-drawing tasks. The cup-holding task included holding a cup with an open handle and a lid. The cup weighed approximately 460 g when filled with water (~80% volume) and with the sensor attached. Participants were asked to sit at a table, lift the filled cup above the table, and stabilize it for approximately 7 s. They performed this task three times, each with a different grip type: (1) around-the-cup grip (holding the cup itself without using the handle), (2) below-the-handle grip (holding the cup under the handle and leaning the handle on their fingers with the thumb on the handle or around the cup), and (3) on-the-handle grip (holding only the handle). The participants were asked to perform the task with the hand they usually use and were allowed to lean their elbow on the table. If they preferred leaning, they were asked to perform all three tasks in the same way to ensure consistency.

**Figure 1:**
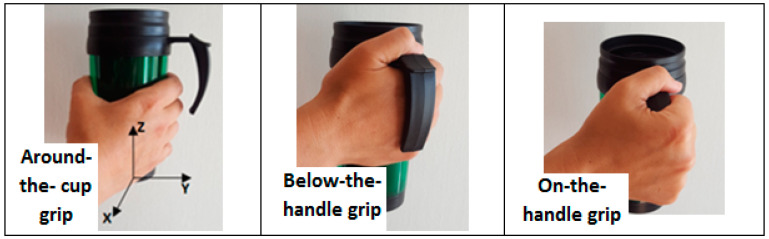
Cup with Axis Orientation of Measured Acceleration and Varied Cup-Grip Types.

A measurement system that included a three-axis accelerometer unit (ADXL335 with a sensitivity of 300 mv/g and range of ±3 g) was attached to the cup while data was sampled at 100 Hz through a data acquisition system (NI-6008) attached to a laptop computer. The sensor was attached to the cup, so that the *X* and *Y* axes (directions) of the measured acceleration were in the plane parallel to the bottom of the cup, and the *Z* axis was the vertical axis (height), as presented in Figure 1.

The spiral drawing task was performed using a digital graphic board with a matching inked pen, and the data from the board included pen-tip coordinates in time. Participants were asked to draw a spiral between lines of a predrawn spiral with an interloop width of 1.5 cm. For more details, refer to [15].

### 2.3. Data Processing

Data from the acceleration measurements were converted from volt units (v) to acceleration units (g), using calibration coefficients of the sensor (in each *X*, *Y*, and *Z* axis). Further analysis used the 5 s from the end of the measurements, which included filtering the signal noise by using a fourth-order Butterworth low pass filter (LPF) with a cutoff frequency of 20 Hz. Time- and frequency-domain analyses were used to analyze tremor features. Most outcome measures were analyzed for each acceleration axis, but measures addressing the contributions of all axes were also calculated for total tremor effect.

In the frequency domain, the following outcome measures were calculated after frequency analysis of the acceleration signal, using the Fast Fourier Transform (FFT) procedure: for the *X*, *Y* and *Z* axes, peak amplitude (Pamp) of the acceleration spectral signal between 4 and 12 Hz, integration of spectral signal ±1 Hz around main frequency, and integration of the spectral signal between 4 and 20 Hz, and total Pamp, calculated as the square root of the sum of Pamp squared in each axis (TPamp = √(Pamp_x_^2^ + Pamp_y_^2^ + Pamp_z_^2^). These outcome measures were based on and addressed in previous research methods using accelerometers and digital board data analysis [13,14,22,32].

In the time domain, we defined additional outcome measures. A filtered signal was calculated using an LPF [33] with a cutoff frequency of 4 Hz and a moving average window of 20 sample points. Deviations of the acceleration from the filtered signal and the sum of square errors/ deviations (SSE) were computed. The SSE was then normalized by the number of sampling points (nSSE): nSSE between acceleration and filtered signal using LPF for the *X*, *Y* and *Z* axes; total deviations, computed as the sum of nSSE from the *X*, *Y* and *Z* axes (TnSSE = nSSE_x_ + nSSE_y_ + nSSE_z_); nSSE between acceleration and filtered signal using a moving average window (width of 20 points) in the *X* and *Y* axes; and mean amplitude computed from averaged consequential peaks for the *X*, *Y* and *Z* axes [16,34]. Because not all frequency-domain analyses resulted in single, clear, main peak amplitude, the last outcome measure was computed to compare the amplitude calculated from FFT.

Our analysis of data from the digital graphic board included normalized SSE of defined outcome measures, computed as the radial and distance curve deviation from a filtered curve (LPF of 4 Hz) [15]. To check the sensitivity of results for the chosen cutoff frequency, we also analyzed the grip-type comparison results for 3 Hz cutoff frequency.

### 2.4. Statistical Analysis 

Statistical analysis included comparison between the ET and control groups using a Mann-Whitney test. We analyzed differences between acceleration axes and the effects of different grip characteristics in the ET group using Freidman and post hoc Wilcoxon tests. Correlations between cup tasks, demographics, general tremor characteristics, and the spiral-drawing task outcome measures were performed using Spearman correlation factors. The statistical analysis was performed using nonparametric tests because there were 20 participants or fewer in each group and most outcome measures were not normally distributed. The statistical analysis was carried out using SPSS software.

## 3. Results

### 3.1. Demographic and General Information

The ET group’s mean MMSE score was 28.8 (*SD* = 1.4), and the control group’s mean was 29.1 (*SD* = 1.2). The ET group was 50% men with a mean age of 64.9 years (*SD* = 15.7; range 23.0–81.7), and the control group consisted of 38.9% men with a mean age of 64.4 years (*SD =* 10.9; range 42.7–78.5). For the ET group, the mean tremor duration since it was noticed was 21.9 years (*SD =* 15.8; range 4.0–55.0), and mean total CADET score was 32.4 (*SD =* 17.5; range 5.8–66.7). Most participants (80% ET group and 88.9% control group) performed the task with their right hand. Demographic features and general information regarding the ET group’s tremor characteristics are described in more detail at [15,19].

### 3.2. Comparing the ET and Control Groups

Significant differences (*p* < 0.05) were found between the research and the control group for all outcome measures and in all grip types and axes (directions). Results comparing the research and control groups for all grips in the *X*, *Y*, and *Z* axes for nSSE between the acceleration and filtered signal outcome measures are presented in Table 1.

All three grip types had significant (*p* < 0.01) differences between the ET and control group participants for outcome measures of nSSE from the filtered signal (below 4 Hz). Results of the group comparison, while changing the cutoff frequency to 3 Hz in nSSE outcome measure calculation, yielded significant results as well.

### 3.3. Comparing Axes Acceleration

Comparing results between axes for the nSSE from filtered signal outcome measures using the Freidman test yielded significant differences for the ET group in all grip types (around-the-cup grip: χ^2^ = 10.9, *p* = 0.004; below-the-handle grip: χ^2^ = 11.2, *p* = 0.004; on-the-handle grip: χ^2^ = 24.3, *p* < 0.001). A comparison of nSSE results for the control group between axes yielded significant differences for only the on-the-handle grip (χ^2^ = 8.44, *p* = 0.015). Results from the post hoc Wilcoxon tests showed values between outcome measures for the ET participants which did not yield a significant result when comparing the *X* and *Y* axes. However, significant (*p* < 0.05) differences were found between the *X* and *Z* axes and the *Y* and *Z* axes. Results of axes comparison and mean values for the outcome measures in the *Y* axis are presented in Table 2 (we present *Y*-axis results because those mean values were higher for most outcome measures).

As shown in Table 2, significant differences (*p* < 0.01 for most results) were found between the *Z* axis and both the *X* and *Y* axes in the ET group, with higher mean values for the *X* and *Y* axes compared to the *Z* axis. Results while changing the cutoff frequency to 3 Hz in the nSSE outcome measure calculation yielded significant results, as well (between *Z* and both *X* and *Y* axes).

### 3.4. Comparing Grip Types

Comparing the results between the three grip types for the ET group for the *X* and *Y* axes, using the Freidman test, yielded significant differences for the *X* axis (but not for the *Y* axis): Pamp was χ^2^ = 13.3, *p* = 0.001; 1 Hz integral was χ^2^ = 13.3, *p* = 0.001, 4 to 20 Hz integral was χ^2^ = 11.2, *p* = 0.004, nSSE was χ^2^ = 15.6, *p* < 0.001; and mean amplitude time domain was χ^2^ = 10.9, *p* = 0.004. Significant differences were also found for the TPamp, χ^2^ = 11.1, *p* = 0.004, and TnSSE χ^2^ = 9.1, *p* = 0.01. Results of the post hoc Wilcoxon tests for these outcome measures in the *X* and *Y* axes yielded no significant differences between the below-the-handle grip and the around-the-cup grip. Nevertheless, there were significant differences between the on-the-handle grip and the below-the-handle grip in the *X* axis and between the on-the-handle grip and the around-the-cup grip in both *X* and *Y* axes (*p* < 0.05 for all outcome measures tested), with mean values higher for the on-the-handle grip.

Regarding the outcome measures of all axes, the TnSSE and TPamp were not significantly different between the on-the-handle and the below-the-handle grips. However, they were significant when comparing the on-the-handle and the around-the-cup grips. Changing the cutoff frequency to 3 Hz in the nSSE outcome measure calculation yielded significant results between the on-the-handle and the around-the-cup grips. These results were still significant after addressing the issue of different thumb positions for the below-the-handle grip and excluding the data when the thumb was not on the handle (remaining *n =* 15).

**Figure 2:**
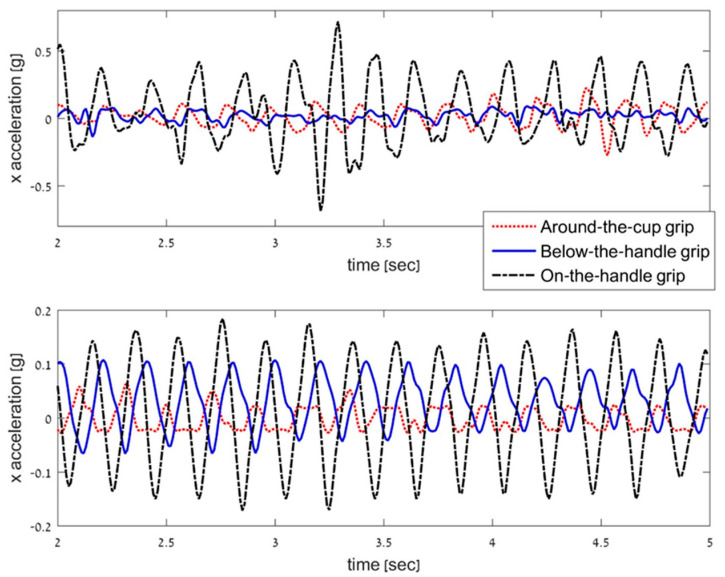
Example of *X*-Axis Acceleration for Two Participants with Essential Tremor.

Analyzing the prevalence of the normalized nSSE values in the different grips, values were higher for 90% of the participants in the *X* axis and for 70% in the *Y* axis for the on-the-handle grip versus the around-the-cup grip. Results showed higher acceleration for the *X* and *Y* axes for 80% and 55% of the participants, respectively, when comparing the on-the-handle and the below-the-handle grips. An example of the *X* axis acceleration signal for two participants with ET in the three grip types is presented in Figure 2.

### 3.5. Correlation Analysis of Cup-Task and Drawing-Task Outcome Measures

Table 3 presents the correlation analysis results for cup outcome measures, age, and spiral deviation from filtered radius curve outcome measure for the *X* and *Y* axes (*r* > 0.3, *p* < 0.05).

From the correlation analysis presented in Table 3, medium to high correlations were found between all cup and spiral outcome measures in the on-the-handle grip. Similar results were found for the around-the-cup grip, except for the Pamp outcome measure, which correlated only in the *Y* axis. Correlations were significant between TnSSE, TPamp, and spirals for all three grip types. The correlations remained significant between most cup and spiral outcome measure, after excluding cases of detachment from paper (resulting in *n =* 18 for ET participants in the spiral task). Medium correlation was found between all cup outcome measures and age in the on-the-handle grip for the *X* or *Y* axis, or both. No significant correlations were found between cup measures and the CADET mean scores or duration of tremor since noticed.

## 4. Discussion

Our research focused on tremor characteristics during a cup-holding task and the effect of grip types on actual performance. The effect of the tremor was evaluated from cup-acceleration measures while expecting no acceleration changes when the cup was stable. Cup-acceleration measures included the amplitude of acceleration curves and its deviation from a filtered acceleration curve, which was defined and computed through several methods. The significant difference for acceleration outcome measures in all axes (direction) and grip types found between the research and control groups supports the feasibility of accelerometer use in tremor measurements. Such usage was implemented in previous research using varied hand-acceleration outcome measures [7,21,30].

Mean values for the peak amplitude of cup acceleration in the ET group were similar to those of previous results measuring finger- and hand-postural acceleration (for a single-axis acceleration measurement) [9,23]. We found no previous results comparing cup acceleration between ET and control groups.

Regarding the difference between axes of measured acceleration for the ET participants when comparing the *X*, *Y*, and *Z* axes, the significant differences found between the *Z* axis and both the *X* and *Y* axes (with lower values in the *Z* axis) may indicate that tremor caused higher instability in those axes. Previous research showed that postural tremor in ET manifested as a more flexion extension movement of the wrist (then supination–pronation) [27]. Because the tremor was transferred to the cup and the hand supinated in 90 degrees, higher acceleration amplitude was expected in the *X* and *Y* axes (bottom surface of the cup plane) in all three grip types. Nevertheless, tremor in other joints or directions may have manifested [27] and affected the results because these other joints or directions were not constrained, that is, due to supination–pronation movement of the wrist or effects of the elbow and shoulder joints (most participants did not lean their elbow on the table) or finger joints (including thumb-joint movements). 

A better understanding of the axis of acceleration and the forces exerted on the object held may help widen the theoretical base for future cup design for people with ET.

When analyzing the effect of grip types on tremor, as measured by cup acceleration, significant differences with higher values were found between the on-the-handle grip and both the below-the-handle and around-the-cup grips in the *X* axis and both the *X* and *Y* axes, respectively. Thus, it seems that the on-the-handle grip had the highest effect on the tremor as measured by cup outcome measures. The three grip types evaluated in this research made users hold the cup in various postures. These postures may have allowed the users to apply the force needed to stabilize the cup against gravity at different cup points and over different areas for each grip. For example, both the around-the-cup and below-the-handle grips allowed the hand to surround the cup. However, in the below-the-handle grip, participants were asked to lean the handle on their fingers to reduce the applied grip force.

The on-the-handle grip causes a different grip aperture by applying force to the handle, resulting in less surface contact. Presumably, the greater the moment of force generated relative to the wrist joint (due to cup weight and the distance from its center of mass to the wrist), the more force which is needed in the wrist muscles to stabilize the cup. From previous research, force levels have been found to affect tremor amplitude [23,28], and the forces in individuals with ET were found to differ from those of controls in precision-grip tasks [35]. Thus, results for higher tremor (measured by cup outcome measures) in the on-the-handle grip may be due to the different kinetic and kinematic characteristics (posture and forces applied). These results are important because using a handle in some cup types may be necessary, for instance while holding a cup filled with a hot drink.

Furthermore, previous studies indicated that people with ET may implement physical modifications to ADL tasks [19] (in process), such as changing hand posture. A better understanding of the grip type effect may help to provide and advise people with ET about personalized methods of performing ADL.

The significant correlation found between all cup and spiral-drawing task outcome measures in the on-the-handle grip and for most outcome measures in the *Y* axis for the other two grips supports the previous results of Bain and colleagues, which also addressed cup and spiral tasks [21]. In their research, significant correlations were found between hand acceleration in posture tasks (converted to displacement), spiral-visual scores, and volume of water spilled in the cup-holding task [21]. These results are important because digital spiral-task measures were previously found to significantly correlate with tremor-severity assessment scores [36]. However, our results did not correlate with the participants’ CADET self-reported disabilities. The CADET disability questionnaire addresses various ADL tasks beyond holding and stabilizing an object (in the air) and provides a subjective perspective of the disability caused by tremor.

Strengths of this study were its ability to address the impact of different directions of cup-acceleration measurements and varied cup-grip types on the actual performance of people with ET. Limitations of this study may include variability of other task-performance characteristics, such as some participants raising the cup to various heights or leaning their arms or elbows on the table, the different orientations and forces the participants’ grips may have implemented (e.g., varied angles of the hand relative to the cup in the around-the-cup grip), and thumb orientation in the on-the-handle grip. Another limitation was that we could not determine whether possible gravitational artifacts in the accelerometer measurements affected the results because we did not include additional measurement systems such as gyroscopes [10].

In summary, our study provides insight into how ET affects the actual performance of ADL tasks and focuses on the acceleration or instability of the object held rather than that of the hand itself. A simpler method for this performance-based test may be to measure cup acceleration, which does not require a sensor attachment on the hand. Our study also addresses the effect of grip type on the suggested acceleration measure results. This may help future development of assistive devices for tremor suppression and personalized supportive functional treatment, leading to improved life quality among people with ET.

## Figures and Tables

**Table 1:** Comparison of Acceleration in the Time-Domain Analysis.

Grip Type	Axis	nSSE from Filtered Signal 10^−5^ [g^2^] M (SD)		Mann–Whitney	
		ET (*n =* 20)	Control (*n =* 18)	*Z*	*p*
Around-the-cup grip	*X*	184.06 (327.53)	7.91 (6.25)	−4.36	<0.001
	*Y*	535.33 (1664.17)	4.58 (3.32)	−4.24	<0.001
	*Z*	71.78 (144.0)	7.42 (3.98)	−3.19	0.001
Below-the-handle grip	*X*	201.13 (452.89)	16.95 (27.37)	−4.24	<0.001
	*Y*	957.22 (2598.85)	16.95 (30.98)	−4.00	<0.001
	*Z*	52.44 (82.59)	15.63 (31.49)	−3.16	0.002
On-the-handle grip	*X*	1048.56 (2762.87)	24.07 (27.51)	−4.36	<0.001
	*Y*	1303.76 (3512.06)	24.11 (41.66)	−4.06	<0.001
	*Z*	280.49 (552.37)	19.36 (31.30)	−2.87	0.004

Note. nSSE = normalized sum of square errors/deviations between acceleration and filtered signal outcome measure in the *X*, *Y*, and *Z* axes for the research and control groups for all grips; g = acceleration units.

**Table 2:** Results of the *Z*–*X* and *Z*–*Y* Axes Comparisons.

	Grip Type	*Y* Axis	Comparing *Z*–*X* Axes	Comparing *Z*–*Y* Axes
		M (*SD*)	(*Z*, *p*)	
Peak amplitude [g]	Around-the-cup grip	0.05 (0.09)	−2.50 *, 0.012	−3.10 **, 0.002
	Below-the-handle grip	0.06 (0.10)	−2.05 *, 0.040	−2.91 **, 0.004
	On-the-handle grip	0.08 (0.13)	−2.84 **, 0.005	−3.92 ***, <0.001
±1 integral around main frequency [g·Hz]	Around-the-cup grip	0.03 (0.05)	−2.50 *, 0.010	−3.10 **, 0.002
	Below-the-handle grip	0.04 (0.06)	−2.24 *, 0.025	−3.40 **, 0.001
	On-the-handle grip	0.05 (0.07)	−3.06 **, 0.002	−3.83 ***, <0.001
4–20 Hz integral [g·Hz]	Around-the-cup grip	0.06 (0.08)	−3.29 **, 0.001	−3.14 **, 0.002
	Below-the-handle grip	0.08 (0.10)	−3.85 ***, <0.001	−3.47 **, 0.001
	On-the-handle grip	0.09 (0.10)	−3.62 ***, <0.001	−3.92 ***, <0.001
nSSE from filtered signal 10^−5^ [g^2^]	Around-the-cup grip	^a^	−2.80 **, 0.005	−3.20 **, 0.001
	Below-the-handle grip	^a^	−2.72 **, 0.006	−3.29 **, 0.001
	On-the-handle grip	^a^	−2.80 **, 0.005	−3.92 ***, <0.001
Mean amplitude computed from consequential (time domain) peaks [g]	Around-the-cup grip	0.06 (0.10)	−2.50 *, 0.012	−2.99 **, 0.003
	Below-the-handle grip	0.08 (0.13)	−2.99 **, 0.003	−2.99 **, 0.003
	On-the-handle grip	0.09 (0.14)	−2.65 **, 0.008	−3.55 ***, <0.001

Note. ^a^ See Table 1. Wilcoxon tests were used for axes outcome measures in the ET group (*n =* 20) and mean values for the *Y* axis. * *p* < 0.05; ** *p* < 0.01; *** *p* < 0.001.

**Table 3:** Correlation Between Cup Outcome Measures, Age, and Spiral Deviation Outcome Measures, for *X* and *Y* Axes and Grip Types.

Domain	Cup Outcome Measure	Grip Type	Axis	Age	Spiral nSSE
Frequency	Peak amplitude [g]	Around-the-cup grip	*X*		
			*Y*	0.45 *	0.60 **
		Below-the-handle grip	*X*		
			*Y*		0.56 *
		On-the-handle grip	*X*	0.48 *	0.59 **
			*Y*	0.45 *	0.64 **
	±1 integral around main frequency [g·Hz]	Around-the-cup grip	*X*		0.45 *
			*Y*		0.76 **
		Below-the-handle grip	*X*		
			*Y*		0.57 *
		On-the-handle grip	*X*	0.57 *	0.71 **
			*Y*		0.68 **
	4–20 Hz integral [g·Hz]	Around-the-cup grip	*X*		0.64 **
			*Y*	0.49 *	0.74 **
		Below-the-handle grip	*X*		0.53 *
			*Y*		0.69 **
		On-the-handle grip	*X*	0.49 *	0.65 **
			*Y*	0.46 *	0.67 **
Time	nSSE from filtered signal [g^2^]	Around-the-cup grip	*X*		0.52 *
			*Y*		0.73 **
		Below-the-handle grip	*X*		
			*Y*		0.61 **
		On-the-handle grip	*X*	0.50 *	0.64 **
			*Y*		0.61 **
	Mean amplitude computed from consequential peaks [g]	Around-the-cup grip	*X*		0.56 *
			*Y*		0.58 **
		Below-the-handle grip	*X*		
			*Y*		0.59 **
		On-the-handle grip	*X*		0.59 **
			*Y*	0.45 *	0.61 **

Note. * *p* < 0.05, ** *p* < 0.01.

## Data Availability

The dataset analyzed in this study is not publicly available due to the privacy of the participants.
